# 1,4-Diazo­niabicyclo­[2.2.2]octane tetra­chloridozincate monohydrate

**DOI:** 10.1107/S1600536809014822

**Published:** 2009-04-25

**Authors:** Fangming Wang

**Affiliations:** a School of Materials Science and Engineering, Jiangsu University of Science and Technology, Zhenjiang 212003, People’s Republic of China

## Abstract

In the title compound, (C_6_H_14_N_2_)[ZnCl_4_]·H_2_O, the crystal packing is governed by an extensive three-dimensional network of N—H⋯Cl, N—H⋯O and O—H⋯Cl hydrogen bonds. The zinc(II) metal centre has a slightly distorted tetra­hedral coordination geometry.

## Related literature

For the applications of ferroelectric materials, see: Fu *et al.* (2007 ▶); Dawber *et al.* (2005 ▶); Haertling (1999 ▶); Scott (2007 ▶). For the properties and structure of a related diaza­bicyclo­[2.2.2]octane (dabco) salt, see: Szafrański *et al.* (2002 ▶).
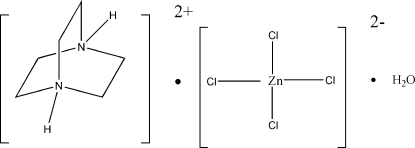


## Experimental

### 

#### Crystal data

(C_6_H_14_N_2_)[ZnCl_4_]·H_2_O
*M* *_r_* = 339.40
Orthorhombic, 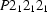

*a* = 8.4483 (17) Å
*b* = 11.705 (2) Å
*c* = 12.976 (3) Å
*V* = 1283.2 (5) Å^3^
*Z* = 4
Mo *K*α radiation
μ = 2.72 mm^−1^
*T* = 291 K
0.30 × 0.28 × 0.26 mm

#### Data collection

Rigaku Mercury2 diffractometer
Absorption correction: multi-scan (*CrystalClear*; Rigaku, 2005 ▶) *T*_min_ = 0.462, *T*_max_ = 0.495
11890 measured reflections
2510 independent reflections
2166 reflections with *I* > 2σ(*I*)
*R*_int_ = 0.053

#### Refinement

*R*[*F* ^2^ > 2σ(*F* ^2^)] = 0.046
*wR*(*F* ^2^) = 0.121
*S* = 1.03
2510 reflections
127 parameters
H-atom parameters constrained
Δρ_max_ = 0.89 e Å^−3^
Δρ_min_ = −0.48 e Å^−3^
Absolute structure: Flack (1983 ▶), 1050 Friedel pairs
Flack parameter: 0.07 (3)

### 

Data collection: *CrystalClear* (Rigaku, 2005 ▶); cell refinement: *CrystalClear*; data reduction: *CrystalClear*; program(s) used to solve structure: *SHELXS97* (Sheldrick, 2008 ▶); program(s) used to refine structure: *SHELXL97* (Sheldrick, 2008 ▶); molecular graphics: *SHELXTL* (Sheldrick, 2008 ▶); software used to prepare material for publication: *SHELXTL*.

## Supplementary Material

Crystal structure: contains datablocks I, global. DOI: 10.1107/S1600536809014822/rz2310sup1.cif

Structure factors: contains datablocks I. DOI: 10.1107/S1600536809014822/rz2310Isup2.hkl

Additional supplementary materials:  crystallographic information; 3D view; checkCIF report

## Figures and Tables

**Table 1:** Hydrogen-bond geometry (Å, °)

*D*—H⋯*A*	*D*—H	H⋯*A*	*D*⋯*A*	*D*—H⋯*A*
N2—H2*C*⋯O1	0.91	1.96	2.809 (8)	154
N1—H1*C*⋯Cl1^i^	0.91	2.64	3.338 (5)	134
N1—H1*C*⋯Cl3^i^	0.91	2.80	3.383 (5)	123
O1—H1*D*⋯Cl3^ii^	0.85	2.82	3.410 (7)	129
O1—H1*E*⋯Cl1^iii^	0.85	2.75	3.454 (7)	141

Symmetry codes: (i) 
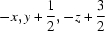
; (ii) 
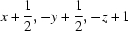
; (iii) 
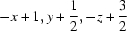
.

**Fig. 1.**
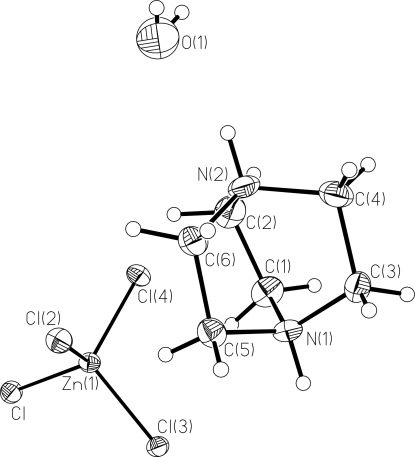
The molecular structure of the title compound, showing the atomic numbering scheme with 30% probability displacement ellipsoids.

**Fig. 2.**
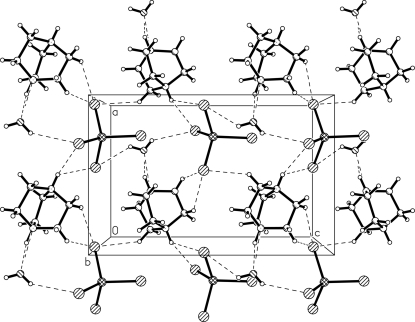
Packing digram of the title compound viewed along the b axis. H-bonding interactions are shown as dashed lines.

## supplementary crystallographic information

### Comment

Ferroelectric materials continue to attract much attention due to their potential applications in memory devices (Fu *et al.*, 2007; Dawber *et al.*, 2005; Haertling, 1999; Scott, 2007). Among these materials, diazabicyclo[2.2.2]octane (dabco) salts with inorganic tetrahedral anions having potassium dihydrophosphate-type (KDP-type) structure have been found to exhibit exceptional dielectric properties (Szafrański *et al.*, 2002). As a contribution to this field, the crystal structure of the title compound is reported here.

The asymmetric unit of the title compound (Fig. 1), contains a doubly protonated C_6_H_14_N_2_^2+^ dication, a ZnCl_4_^2-^ dianion and a water molecule. The zinc(II) metal displays a slightly distorted tetrahedral coordination geometry. In the cation, the protonated N1 atom interacts *via* a bifurcated hydrogen bond with two Cl atoms of a neighbouring anion, while the N2 atom is hydrogen-bonded to a water molecule (Table 1). The water molecule acts as double hydrogen-bond donor to Cl atoms, resulting in an extensive three-dimensional H-bonding network (Fig. 2).

### Experimental

Single crystals of the title compound suitable for X-ray analysis were obtained by slow evaporation at room temperature of a HCl solution (0.5 M) containing diazabicyclo[2.2.2]octane (112 mg) and ZnCl_2_.2H_2_O (172 mg) in an approximate 1:1 molar ratio.

### Refinement

All H atoms were placed in calculated positions, with O—H = 0.85 Å, N—H = 0.91 Å, C—H = 0.97 Å, and refined using a riding model approximation, with *U*_iso_  = 1.2*U*eq(C, N) or 1.5*U*eq(O).

### Crystal data

**Table:**

(C_6_H_14_N_2_)[ZnCl_4_]·H_2_O	*F*(000) = 688
*M**_r_* = 339.40	*D*_x_ = 1.757 Mg m^−^^3^
Orthorhombic, *P*2_1_2_1_2_1_	Mo *K*α radiation, λ = 0.71073 Å
Hall symbol: P 2ac 2ab	Cell parameters from 11517 reflections
*a* = 8.4483 (17) Å	θ = 3.1–27.5°
*b* = 11.705 (2) Å	µ = 2.72 mm^−^^1^
*c* = 12.976 (3) Å	*T* = 291 K
*V* = 1283.2 (5) Å^3^	Block, colourless
*Z* = 4	0.30 × 0.28 × 0.26 mm

### Data collection

**Table:**

Rigaku Mercury2 diffractometer	2510 independent reflections
Radiation source: fine-focus sealed tube	2166 reflections with *I* > 2σ(*I*)
graphite	*R*_int_ = 0.053
Detector resolution: 13.6612 pixels mm^-1^	θ_max_ = 26.0°, θ_min_ = 3.1°
ω scans	*h* = −10→10
Absorption correction: multi-scan (*CrystalClear*; Rigaku, 2005)	*k* = −14→14
*T*_min_ = 0.462, *T*_max_ = 0.495	*l* = −16→16
11890 measured reflections	

### Refinement

**Table:**

Refinement on *F*^2^	Secondary atom site location: difference Fourier map
Least-squares matrix: full	Hydrogen site location: inferred from neighbouring sites
*R*[*F*^2^ > 2σ(*F*^2^)] = 0.046	H-atom parameters constrained
*wR*(*F*^2^) = 0.121	*w* = 1/[σ^2^(*F*_o_^2^) + (0.0665*P*)^2^ + 1.4482*P*] where *P* = (*F*_o_^2^ + 2*F*_c_^2^)/3
*S* = 1.03	(Δ/σ)_max_ = 0.001
2510 reflections	Δρ_max_ = 0.89 e Å^−^^3^
127 parameters	Δρ_min_ = −0.48 e Å^−^^3^
0 restraints	Absolute structure: Flack (1983), 1050 Friedel pairs
Primary atom site location: structure-invariant direct methods	Flack parameter: 0.07 (3)

### Special details

**Table:**

Geometry. All e.s.d.'s (except the e.s.d. in the dihedral angle between two l.s. planes) are estimated using the full covariance matrix. The cell e.s.d.'s are taken into account individually in the estimation of e.s.d.'s in distances, angles and torsion angles; correlations between e.s.d.'s in cell parameters are only used when they are defined by crystal symmetry. An approximate (isotropic) treatment of cell e.s.d.'s is used for estimating e.s.d.'s involving l.s. planes.
Refinement. Refinement of *F*^2^ against ALL reflections. The weighted *R*-factor *wR* and goodness of fit *S* are based on *F*^2^, conventional *R*-factors *R* are based on *F*, with *F* set to zero for negative *F*^2^. The threshold expression of *F*^2^ > σ(*F*^2^) is used only for calculating *R*-factors(gt) *etc*. and is not relevant to the choice of reflections for refinement. *R*-factors based on *F*^2^ are statistically about twice as large as those based on *F*, and *R*- factors based on ALL data will be even larger.

### Fractional atomic coordinates and isotropic or equivalent isotropic displacement parameters (Å^2^)

**Table:**

	*x*	*y*	*z*	*U*_iso_*/*U*_eq_	
C1	0.2122 (7)	0.3775 (6)	0.3733 (5)	0.0434 (17)	
H1A	0.1610	0.3189	0.4143	0.052*	
H1B	0.2347	0.4422	0.4177	0.052*	
C2	0.3645 (8)	0.3316 (7)	0.3286 (5)	0.0489 (18)	
H2A	0.3676	0.2490	0.3347	0.059*	
H2B	0.4547	0.3632	0.3650	0.059*	
C3	0.1700 (9)	0.5158 (6)	0.2364 (5)	0.0415 (16)	
H3A	0.1655	0.5809	0.2826	0.050*	
H3B	0.1064	0.5330	0.1761	0.050*	
C4	0.3402 (8)	0.4934 (6)	0.2046 (6)	0.0474 (18)	
H4A	0.3568	0.5167	0.1336	0.057*	
H4B	0.4123	0.5361	0.2482	0.057*	
C5	0.0884 (7)	0.3177 (5)	0.2132 (5)	0.0371 (15)	
H5A	0.0055	0.3356	0.1641	0.045*	
H5B	0.0602	0.2477	0.2487	0.045*	
C6	0.2468 (8)	0.3030 (5)	0.1574 (4)	0.0405 (14)	
H6A	0.2745	0.2227	0.1536	0.049*	
H6B	0.2394	0.3328	0.0878	0.049*	
Cl1	0.20057 (18)	−0.18534 (13)	1.07634 (13)	0.0420 (4)	
Cl2	0.2573 (2)	−0.03346 (13)	0.81713 (10)	0.0444 (4)	
Cl3	0.03672 (18)	0.09791 (13)	1.03379 (13)	0.0371 (4)	
Cl4	0.47943 (19)	0.06684 (14)	1.03500 (14)	0.0414 (4)	
N1	0.1070 (6)	0.4132 (4)	0.2888 (4)	0.0308 (11)	
H1C	0.0103	0.4303	0.3156	0.037*	
N2	0.3689 (6)	0.3664 (5)	0.2161 (5)	0.0444 (15)	
H2C	0.4659	0.3493	0.1899	0.053*	
O1	0.6587 (8)	0.2561 (5)	0.1794 (5)	0.086 (2)	
H1D	0.6849	0.2659	0.1167	0.128*	
H1E	0.7293	0.2845	0.2184	0.128*	
Zn1	0.24738 (9)	−0.01799 (5)	0.99321 (4)	0.0333 (2)	

### Atomic displacement parameters (Å^2^)

**Table:**

	*U*^11^	*U*^22^	*U*^33^	*U*^12^	*U*^13^	*U*^23^
C1	0.043 (4)	0.054 (4)	0.033 (3)	0.007 (3)	−0.009 (3)	−0.004 (3)
C2	0.042 (4)	0.052 (4)	0.052 (4)	0.006 (3)	−0.020 (3)	−0.008 (4)
C3	0.044 (4)	0.031 (3)	0.050 (4)	0.005 (3)	0.005 (3)	0.003 (3)
C4	0.036 (4)	0.054 (4)	0.053 (4)	−0.011 (3)	0.012 (3)	−0.010 (4)
C5	0.038 (3)	0.037 (4)	0.037 (3)	−0.003 (3)	−0.004 (3)	0.003 (3)
C6	0.034 (3)	0.043 (3)	0.045 (3)	−0.007 (3)	−0.007 (3)	−0.018 (3)
Cl1	0.0443 (9)	0.0383 (8)	0.0435 (8)	0.0001 (7)	0.0069 (6)	0.0048 (7)
Cl2	0.0477 (9)	0.0536 (9)	0.0318 (7)	−0.0048 (9)	0.0041 (8)	−0.0058 (6)
Cl3	0.0322 (8)	0.0407 (8)	0.0384 (9)	0.0023 (6)	0.0004 (7)	−0.0041 (7)
Cl4	0.0368 (8)	0.0459 (8)	0.0414 (9)	−0.0079 (7)	−0.0055 (7)	0.0029 (8)
N1	0.022 (2)	0.042 (3)	0.029 (3)	0.004 (2)	0.0009 (19)	−0.002 (2)
N2	0.022 (3)	0.051 (3)	0.061 (4)	0.003 (2)	0.004 (2)	−0.018 (3)
O1	0.067 (4)	0.083 (5)	0.106 (5)	0.004 (4)	−0.014 (4)	−0.001 (4)
Zn1	0.0317 (3)	0.0375 (3)	0.0307 (3)	−0.0011 (3)	0.0009 (3)	0.0002 (3)

### Geometric parameters (Å, °)

**Table:**

C1—N1	1.472 (7)	C5—C6	1.531 (9)
C1—C2	1.510 (9)	C5—H5A	0.9700
C1—H1A	0.9700	C5—H5B	0.9700
C1—H1B	0.9700	C6—N2	1.482 (8)
C2—N2	1.515 (9)	C6—H6A	0.9700
C2—H2A	0.9700	C6—H6B	0.9700
C2—H2B	0.9700	Cl1—Zn1	2.2710 (17)
C3—N1	1.479 (8)	Cl2—Zn1	2.2936 (15)
C3—C4	1.519 (9)	Cl3—Zn1	2.2989 (17)
C3—H3A	0.9700	Cl4—Zn1	2.2635 (17)
C3—H3B	0.9700	N1—H1C	0.9100
C4—N2	1.514 (9)	N2—H2C	0.9100
C4—H4A	0.9700	O1—H1D	0.8499
C4—H4B	0.9700	O1—H1E	0.8500
C5—N1	1.496 (8)		
			
N1—C1—C2	109.2 (5)	C6—C5—H5B	110.2
N1—C1—H1A	109.8	H5A—C5—H5B	108.5
C2—C1—H1A	109.8	N2—C6—C5	108.0 (5)
N1—C1—H1B	109.8	N2—C6—H6A	110.1
C2—C1—H1B	109.8	C5—C6—H6A	110.1
H1A—C1—H1B	108.3	N2—C6—H6B	110.1
C1—C2—N2	107.2 (5)	C5—C6—H6B	110.1
C1—C2—H2A	110.3	H6A—C6—H6B	108.4
N2—C2—H2A	110.3	C1—N1—C3	110.8 (5)
C1—C2—H2B	110.3	C1—N1—C5	109.9 (5)
N2—C2—H2B	110.3	C3—N1—C5	110.1 (5)
H2A—C2—H2B	108.5	C1—N1—H1C	108.7
N1—C3—C4	109.0 (5)	C3—N1—H1C	108.7
N1—C3—H3A	109.9	C5—N1—H1C	108.7
C4—C3—H3A	109.9	C6—N2—C4	109.2 (5)
N1—C3—H3B	109.9	C6—N2—C2	110.1 (5)
C4—C3—H3B	109.9	C4—N2—C2	110.8 (5)
H3A—C3—H3B	108.3	C6—N2—H2C	108.9
N2—C4—C3	107.1 (5)	C4—N2—H2C	108.9
N2—C4—H4A	110.3	C2—N2—H2C	108.9
C3—C4—H4A	110.3	H1D—O1—H1E	109.5
N2—C4—H4B	110.3	Cl4—Zn1—Cl1	114.55 (7)
C3—C4—H4B	110.3	Cl4—Zn1—Cl2	103.99 (7)
H4A—C4—H4B	108.5	Cl1—Zn1—Cl2	114.29 (6)
N1—C5—C6	107.6 (5)	Cl4—Zn1—Cl3	110.90 (6)
N1—C5—H5A	110.2	Cl1—Zn1—Cl3	105.40 (6)
C6—C5—H5A	110.2	Cl2—Zn1—Cl3	107.63 (7)
N1—C5—H5B	110.2		

### Hydrogen-bond geometry (Å, °)

**Table:**

*D*—H···*A*	*D*—H	H···*A*	*D*···*A*	*D*—H···*A*
N2—H2C···O1	0.91	1.96	2.809 (8)	154
N1—H1C···Cl1^i^	0.91	2.64	3.338 (5)	134
N1—H1C···Cl3^i^	0.91	2.80	3.383 (5)	123
O1—H1D···Cl3^ii^	0.85	2.82	3.410 (7)	129
O1—H1E···Cl1^iii^	0.85	2.75	3.454 (7)	141

Symmetry codes: (i) −*x*, *y*+1/2, −*z*+3/2; (ii) *x*+1/2, −*y*+1/2, −*z*+1; (iii) −*x*+1, *y*+1/2, −*z*+3/2.
